# A Wide-Range, Highly Stable Intelligent Flexible Pressure Sensor Based on Micro-Wrinkled SWCNT/rGO-PDMS with Efficient Thermal Shrinkage

**DOI:** 10.3390/bios15020122

**Published:** 2025-02-19

**Authors:** Lei Fan, Zhaoxin Wang, Tao Yang, Qiang Zhao, Zhixin Wu, Yijie Wang, Xue Qi, Lei Zhang

**Affiliations:** 1 Key Laboratory of Micro/Nano Devices and Systems, Ministry of Education, North University of China, Taiyuan 030051, China; wzx1132138479@163.com (Z.W.); 18003415061@163.com (T.Y.); 13073569347@163.com (Q.Z.); wuzhixin0513@163.com (Z.W.); 19834401268@163.com (Y.W.); 20230103@nuc.edu.cn (X.Q.); 20210022@nuc.edu.cn (L.Z.); 2 State Key Laboratory of Dynamic Measurement Technology, North University of China, Taiyuan 030051, China

**Keywords:** thermal shrinkage, micro-wrinkled structure, wide-range, highly stable, flexible pressure sensor

## Abstract

Flexible pressure sensors have drawn growing attention in areas like human physiological signal monitoring and human–computer interaction. Nevertheless, it still remains a significant challenge to guarantee their long-term stability while attaining a wide detection range, a minute pressure testing limit, and high sensitivity. Inspired by the wrinkles on animal skins, this paper introduces a flexible pressure sensor with wrinkled microstructures. This sensor is composed of a composite of reduced graphene oxide (rGO), single-walled carbon nanotubes (SWCNTs), and polydimethylsiloxane (PDMS). After optimizing the proportion of the composite materials, the flexible pressure sensor was manufactured using highly efficient heat-shrinkable films. It has a sensitivity as high as 15.364 kPa^−1^. Owing to the wrinkled microstructures, the sensor can achieve an ultra-wide pressure detection range, with the maximum reaching 1150 kPa, and is capable of detecting water wave vibrations at the minimum level. Moreover, the wrinkled microstructures were locked by PDMS. The sensor acquired waterproof performance and its mechanical stability was enhanced. Even after 18,000 cycles of repeated loading and unloading, its performance remained unchanged. By combining with an artificial neural network, high-precision recognition of different sounds and postures when grasping different objects was realized, with the accuracies reaching 98.3333% and 99.1111%, respectively. Through the integration of flexible WIFI, real-time wireless transmission of sensing data was made possible. In general, the studied sensor can facilitate the application of flexible pressure sensors in fields such as drowning monitoring, remote traditional Chinese medicine, and intelligent voice.

## 1. Introduction

Novel flexible sensors can be used in wearable electronic products, biomedicine, and electronic skin [1,2,3,4,5]. Flexible pressure sensors have been under extensive research owing to their diverse applications in human motion and health monitoring. For instance, pressure sensors are capable of detecting human pulse to supply data for healthcare [6]. They can also be affixed near the human larynx to capture vocal cord vibrations for voice recognition [7] and integrated with the Internet of Things to identify human movements or grasping actions [8]. Nevertheless, all these applications mandate that the sensors possess excellent minute pressure detection capabilities and high stability for long-term use in human body monitoring. Moreover, wide-range sensors can accommodate large-angle bending of joints during vigorous human movements.

Researchers have put forward a variety of microstructures to enhance the performance of pressure sensors, including interlocking structures [9], wrinkles [10], cracks [11], and pyramid arrays [12]. Yong-song Tan showcased a hierarchical interlocking structure with a cylindrical array. By using a cylindrical array fabricated from polyvinyl alcohol (PVA) and polyaniline (PANI), they were able to enlarge the contact area of single-walled carbon nanotubes (SWCNTs), thus bolstering electrical conductivity. Moreover, voids were introduced into the sensing layer, which served to cut down the hysteresis time resulting from the viscoelasticity of materials, speed up the response, and widen the sensor’s detection range [9]. Tao Wang devised a micro-pyramid array structure [12]. When substantial pressure was exerted, this micro-pyramid array structure would deform conspicuously, augmenting the contact area among conductive materials. In comparison with flat film electrodes, the sensitivity of the film electrodes featuring the micro-pyramid array structure was tripled. D Dong introduced a micro-crack structure [11]. When it was compressed by an external strain, the crack structure would close, leading to an increase in the contact area within the conductive path and the formation of more local conductive paths. This made the structure more responsive to the external compressive strain.

As a two-dimensional carbon material, graphene possesses excellent electrical and mechanical properties. Its derivatives, such as graphene oxide, reduced graphene oxide, carbon nanotubes, and carbon black, are also commonly used as conductive materials for flexible sensors. Jin Jia et al. fabricated a film with a hierarchical wrinkled structure similar to the surface of human skin based on reduced graphene oxide (rGO) for pressure measurement. It can respond to a pressure as low as 42 Pa [13], but its measurement range only reaches up to 3 KPa at most. Kim et al. prepared a fabric pressure sensor using single-walled carbon nanotubes and reduced graphene oxide, which has high mechanical stability and flexibility, that was used for detecting human movements [14]. However, its ability to detect minute pressure is rather weak. Han et al. utilized carbon black and dry paper to create a highly sensitive flexible pressure sensor with a sensitivity of up to 51.23 kPa^−1^, capable of detecting both slight and large pressures [15]. Nevertheless, due to its paper-based material, it has poor temperature stability and waterproof performance. In the face of the above-mentioned problems, polydimethylsiloxane (PDMS), as an organic elastomer, has excellent biocompatibility and waterproof properties. It has been widely used in flexible sensors [16]. To some extent, it has improved the performance of sensors and has also led to the development of many new types of sensors based on PDMS. Mainly, composite materials are constructed by mixing conductive fillers with PDMS or coating PDMS on conductive materials. Yang et al. reported a wide-range pressure sensor. Based on the principle that NaCl dissolves in water, they mixed PDMS with NaCl and then dried it. By continuously centrifuging it in deionized water to remove NaCl, they obtained a porous composite material with a working range of up to 1275 KPa [17]. However, the sensitivity just up to 1.412 KPa^−1^. Sadiq et al. fabricated a three-layer flexible pressure sensor, in which the conductive film was sandwiched between two PDMS protective films to prevent the conductive film from cracking, and it could sense the tiny movements of facial expressions [18]. Although the above-mentioned sensors can already be applied to human physiological monitoring, there are still problems such as a small measurement range, low stability, poor waterproof performance, and weak micro-pressure detection ability. Therefore, how to obtain a pressure sensor that simultaneously takes into account a wide measurement range, good micro-pressure detection ability, high stability, and excellent waterproof performance remains a major challenge.

In this study, we put forward a flexible pressure sensor predicated on SWCNT/rGO-PDMS, which is characterized by excellent minute pressure detection capabilities, high stability, a broad measurement range, and outstanding waterproof properties. The incorporation of a reduced graphene oxide–carbon nanotube composite material has significantly enhanced the sensitivity. In contrast to using rGO alone, the SWCNTs added to rGO are interwoven across the rGO surface. Their tubular architecture facilitates the flow of carriers through the various layers of rGO, thereby diminishing the overall resistance of the sensor and endowing SWCNT/rGO with heightened sensitivity. Furthermore, through an in-depth investigation of SWCNT/rGO composite materials with diverse compositions, we ascertained the optimal proportion of SWCNT to rGO as 7:3. The micro-wrinkle structure was fabricated via a simple polystyrene heat shrinkage technique, effectively broadening the pressure sensing range [19]. The maximum detectable pressure can soar up to 1150 KPa. By coating with PDMS for phase locking, the sensor is empowered to detect even the smallest pressure fluctuations caused by water wave vibrations while maintaining robust stability. It has been proven to remain unfaltering after enduring over 18,000 pressure cycles at 16.75 KPa. Owing to its wide pressure measurement range, high sensitivity, and remarkable minute pressure detection performance, when combined with the Internet of Things and artificial intelligence, we have further validated its applications in human physiological signal monitoring, water wave detection, voice recognition, and object grasping. The developed sensor evidently holds distinct advantages in wearable devices, human–computer interaction, and drowning monitoring.

## 2. Materials and Methods

### 2.1. Materials

For this experiment, we used graphite powder and SWNTs (Nanjing, China, XFNANO Materials Tech Co., Ltd.), NaNO_3_ (Guangdong, China, FULIN, 0.1 mol/L), potassium permanganate (KMnO_4_, Shanghai, China, Sigma-Aldrich, ≥99%), hydrogen peroxide (H_2_O_2_, Shanghai, China, Sigma-Aldrich, 3 wt%), polydimethylsiloxane (PDMS, Sylgard 184, Midland, MI, USA, Dow Corning), n-hexane (99%), ethanol (Shanghai, China, Aladdin, 99%), polystyrene (PS, average MW is 192,000, Hangzhou, China, Alibaba), dichloromethane (Shanghai, China, MACKLIN), ascorbic acid (Hunan, China, BKMAM), polyimide and Cu (Hangzhou, China, Alibaba), and deionized (DI) water.

### 2.2. Fabrication of SWCNT/GO Composite Films with Micro-Wrinkle Structure

Graphene oxide was synthesized using the Hummers method [20], described in the Supplementary Materials. Firstly, a single-walled carbon nanotube solution with a concentration of 0.1 mg·mL^−1^ was blended with the graphene oxide solution at a weight ratio of GO:SWNT = 1:1. Subsequently, the mixture underwent ultrasonic treatment for 20 min and was then vacuum-filtered to obtain the SWCNT/GO composite film. After drying in an oven set at 45 °C, the film was immersed in absolute ethanol to be peeled off the filter paper. Next, the polystyrene (PS) heat-shrinkable film was cut into 5 cm × 5 cm square pieces. These pieces were washed sequentially with deionized water and absolute ethanol and then treated with plasma to augment the surface adhesion of the PS. The peeled SWCNT/GO composite film was then transferred onto the PS heat-shrinkable film and allowed to dry naturally for 1 h. Finally, it was placed in a vacuum oven at 135 °C for 15 min of heat shrinkage, after which the SWCNT/GO composite film with micro-wrinkled structures was retrieved.

### 2.3. Preparation of SWCNT/rGO-PDMS Sensors

**Figure 1:**
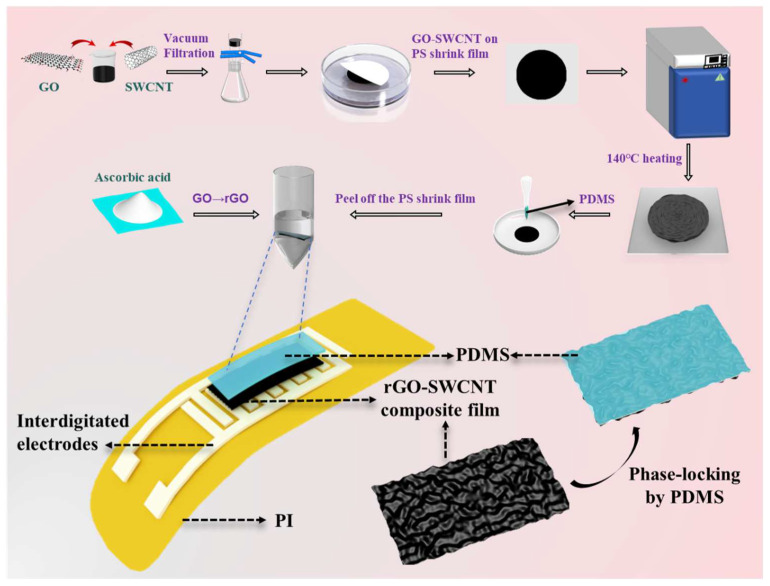
Fabrication of SWCNT/rGO-PDMS sensor.

The SWCNT/GO composite film was placed on a spin coater. Subsequently, the prepared PDMS (with a weight ratio of prepolymer to curing agent of 10:1) was spin-coated onto the surface of the composite film by a step of 100 rpm @ 10 s and 500 rpm @ 20 s. Spin-coating was carried out 3 times to ensure that the PDMS formed a uniform thin layer. After drying in an oven set at 100 °C, the sample was immersed in dichloromethane to dissolve the heat-shrinkable film. Eventually, 50 mg/mL of ascorbic acid was utilized to chemically reduce GO to rGO, yielding the SWCNT/rGO-PDMS film. The composite SWCNT/rGO-PDMS film was then cut to the appropriate size. Then, it was laid over the interdigital electrodes and encapsulated with Pu tape. PU tape has waterproof and breathable properties, which can better fit the sensor to the skin. Wires were connected to both ends of the interdigital electrodes. In this way, as illustrated in Figure 1, a flexible pressure sensor was obtained which consisted of PDMS on the upper layer, SWCNT/rGO film in the middle layer, and PI film with interdigital electrodes on the lower layer. PI electrodes were created by direct ink writing with polyimide and Cu.

### 2.4. Characterization and Measurement

Raman spectroscopy signals can directly display the stacking degree and functionality of graphene materials. The conjugation degree of the GO structure during the reduction process can be deduced from the ratio of the D peak to the G peak in the Raman spectrum. Raman spectroscopy is employed to examine whether the sensitive material, rGO, has been successfully reduced, coexists with carbon nanotubes, and shows no significant agglomeration. The micro-wrinkled structures on the surface of the composite film samples are analyzed using scanning electron microscopy (SEM, Tokyo, Japan, Hitachi S-4800, 5 kV). Pressure tests on the sensor are carried out with a pressure gauge (ZQ-32, Guangdong, China, zhiqu) and a tensile testing machine. Under a 1 V direct current power supply, a high-precision digital multimeter (KEYSIGHT34465A, Beijing, China, Keysight Technologies) is utilized to record the output current data of the sensor.

## 3. Results and Discussion

### 3.1. Characterization of SWCNT/rGO-PDMS Composite Films

**Figure 2:**
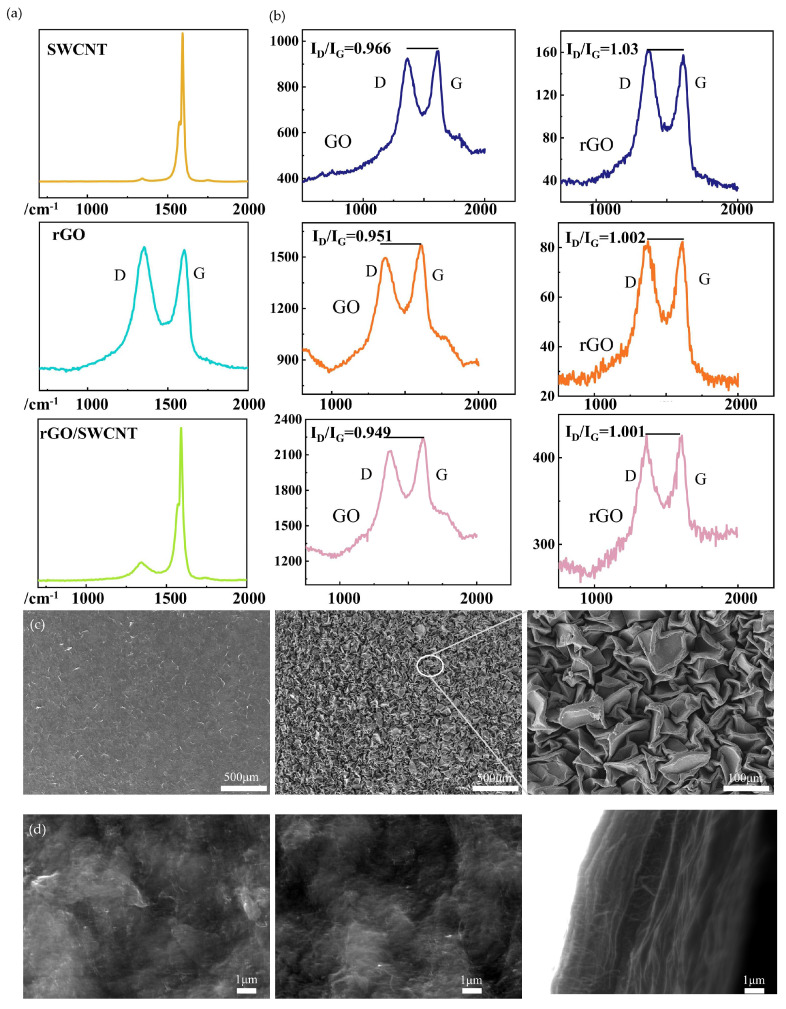
Morphology and characterization of composite film. (**a**) Raman spectra of films of SWCNTs, rGO, and the composite. (**b**) Raman spectra of GO and rGO. (**c**) SEM images of GO-SWCNT composite film before and after heat shrinkage contrast. (**d**) SWCNT distribution in composite film.

The crux of the characterization resides in verifying the success of the rGO reduction reaction and the presence of the wrinkled structure. As depicted in Figure 2a, the signals of SWCNT are observable at 1592 cm^−1^ and 1574 cm^−1^, while the D peak and G peak of rGO are positioned at 1350 cm^−1^ and 1600 cm^−1^, respectively. The Raman spectrum of the composite material concurrently exhibits the waveforms representative of both SWCNT and rGO, which unmistakably attests to their coexistence. Figure 2b presents three sets of Raman spectra acquired before and after the reduction process. Noticeably, the ratio of the D peak to the G peak of rGO surges after reduction, providing conclusive evidence that GO has been effectively converted into rGO. In Figure 2c, the SEM images of the composite film, both prior to and following heat shrinkage, are showcased. These images vividly illustrate the emergence of the wrinkled structure, which consequentially augments the contact area. Figure 2d presents an SEM micrograph of the composite film. The cross-sectional view discloses that SWCNT traverses the rGO surface to forge a conductive network. Notably, the carbon nanotubes no longer aggregate in bundles but are instead dispersed across the rGO sheets. TEM images of rGO/SWCNT mixture in Figure S8 further illustrate the dispersion of SWCNTs. It is tentatively hypothesized that the graphite π–π interaction is responsible for this dispersion of SWCNTs [21].

### 3.2. Sensing Performance of SWCNT/rGO-PDMS

**Figure 3:**
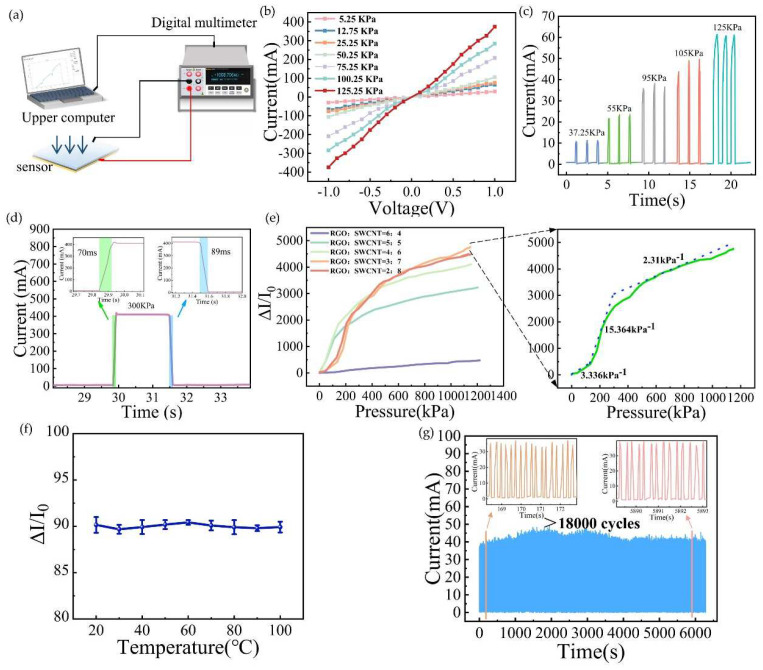
Sensing performance of the SWCNT/rGO pressure sensor. (**a**) Testing platform. (**b**) I-V curve of piezoresistive sensor. (**c**) Current change of the sensor subjected to cyclic pressure (37.25–125 kPa) at the same frequency. (**d**) Sensor response time test. (**e**) Comparison of sensitivity at different material ratios and optimal proportioning sensitivity. (**f**) Stability of the sensor response at various temperatures. (**g**) Sensor repeatability test after 18,000 loading and unloading cycles.

As the pressure increases, the micro-wrinkled structure of the sensor is gradually compressed into a flat shape, enhancing the SWCNT interconnection and reducing the gap between the reduced graphene oxide layers, thereby reducing the resistance. Figure 3a depicts a schematic of the testing system for the piezoresistive pressure sensor. The corresponding test platform is illustrated in Figure S3 of the Supplementary Materials. In order to assess the performance characteristics of the SWCNT/rGO-PDMS sensor in pressure detection, volt–ampere characteristics under seven distinct pressures were tested. As illustrated in Figure 3b, the results manifested a linear trend, signifying an excellent pressure response. Subsequently, repeated tests were carried out on the responses under varying pressures. It was observed in Figure 3c that, under the same pressure level, the responses remained essentially consistent each time, which attested to the sensor’s ability to produce stable output signals. SWCNT/rGO-PDMS exhibited a swift response to external pressure, with a response time of 70 ms and a recovery time of 89 ms, as depicted in Figure 3d. Through an investigation into the sensitivities of different ratios between SWCNT and rGO, an optimal ratio of 7:3 for SWCNT to rGO was ultimately determined to fabricate the composite film. The sensitivity of the sensor could be segmented into three zones—3.336 KPa^−1^ within the low-pressure range (0–132 KPa), 15.364 KPa^−1^ within the medium-pressure range (132–300 KPa), and 2.31 KPa^−1^ within the high-pressure range (300–1150 KPa)—as shown in Figure 3e. When compared to the graphene oxide/carbon nanotube composite, reduced graphene oxide exhibits slightly lower sensitivity than graphene oxide at pressures below 150 kPa. Nevertheless, within the pressure interval spanning from 150 kPa to 500 kPa, the sensor fabricated with reduced graphene oxide/carbon nanotubes demonstrates a sensitivity 1.5-fold that of the sensor made from graphene oxide. The sensitivities corresponding to the diverse material ratios of graphene oxide and carbon nanotubes are illustrated in Figure S6 of the Supplementary Materials. Such a wide measurement range positions the sensor favorably for applications in areas like smart insoles and joint flexion monitoring.

To evaluate whether the sensor could maintain stable output after a temperature increase, it was placed in thermostats set at 20 °C, 30 °C, 40 °C, 50 °C, 60 °C, 70 °C, 80 °C, 90 °C, and 100 °C for one hour each. Thereafter, the sensor was pressed with the same pressure (42 KPa) and the output signals were collected. As shown in Figure 3f, the value of ΔI/I remained largely stable without any significant fluctuations, suggesting that the sensor boasted good temperature stability. After subjecting the sensor to 18,000 loading and unloading cycles with a pressure of 16.75 KPa, the current value in Figure 3f stayed steady around 35 mA without major oscillations, indicating that the sensor possessed high stability.

### 3.3. Monitoring of Human Physiological Signals Using SWCNT/rGO-PDMS

**Figure 4:**
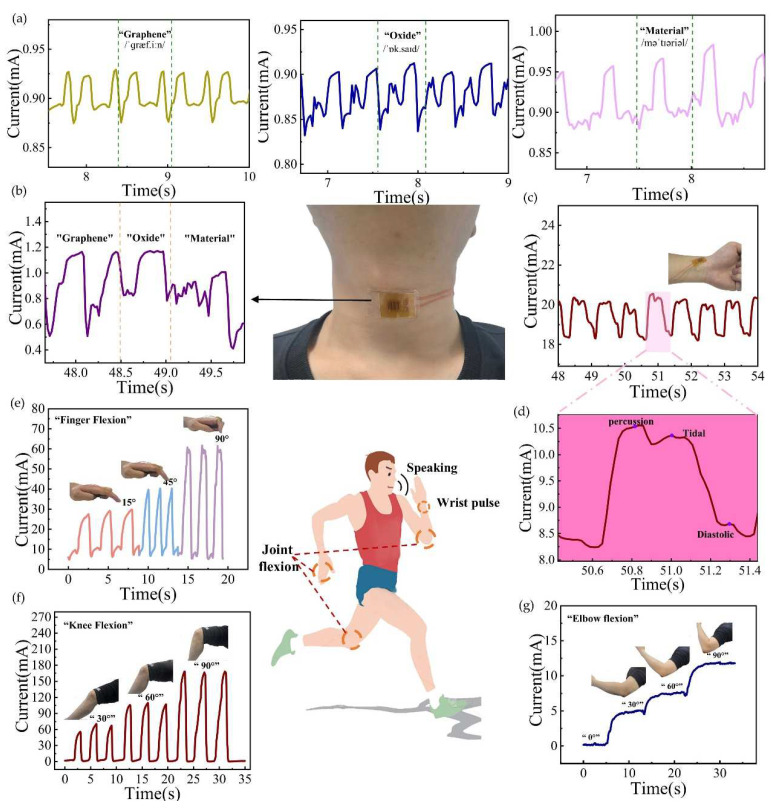
Monitoring results of human physiological signals. (**a**) Sound recognition by a flexible strain sensor attached to the human throat. (**b**) Real-time current response signals induced by vocal fold vibrations while speaking the phrase “graphene oxide material”. (**c**,**d**) Complete cycle of the pulse wave. (**e**) Electrical response when the finger is bent repeatedly. (**f**) Electrical response when the knee is bent repeatedly. (**g**) Change in the current as the elbow is bent at different angles.

The wide detection range, rapid response and recovery capabilities, high sensitivity, and high stability of SWCNT/rGO-PDMS have paved the way for its extensive application in detecting a diverse array of human body signals. To further probe into its efficacy in this regard, the sensors were affixed to different body parts of healthy adult male volunteers, with the aim of capturing the pressure signals generated during physical movements. Specifically, when the sensors were connected beneath the volunteers’ throats, they were able to detect the alterations in pressure signals stemming from vocal cord vibrations and muscle contractions. As depicted in Figure 4a, when the volunteers enunciated the words “Graphene”, “Oxide”, and “Material”, respectively, the sensors picked up distinct pressure signals. Taking the pronunciation of the word “Graphene” as an illustration, the magnitude of the pressure signals received by the sensor remained strikingly consistent each time it was uttered. Figure 4b showcases the pressure signals detected by the sensor when the three words were pronounced in succession. Given the nuanced differences in pronunciation between consecutive words and single-word enunciation, the waveforms of the pressure signals also exhibited slight disparities. Remarkably, by leveraging the pressure signals associated with the pronunciation of individual words, a rough differentiation among the three words could be achieved. This strongly suggests that SWCNT/rGO-PDMS holds significant potential for application in voice recognition.

In addition, this sensor proves its mettle in pulse monitoring. When connected near the radial artery at the wrist and firmly adhered to the skin surface with medical tape under a pre-applied pressure, it can adeptly capture the minute pressure fluctuations of pulse vibrations. Figure 4c presents the real-time pulse data of a volunteer as measured by the sensor. Figure 4d zooms in on the waveform of a single pulse signal, revealing three characteristic peaks—Percussion, Tidal, and Diastolic—which align perfectly with the pulse waveforms observed in clinical electrocardiograms. This finding firmly establishes the sensor’s viability for use in wearable devices related to human health. The sensor’s wide pressure detection range and remarkable stability also render it suitable for attachment to various joint locations on the body to gauge the degree of joint flexion. Figure 4e illustrates the pressure signals corresponding to different finger-bending angles. Notably, as the angle of flexion increases, so does the associated current value. Figure 4f depicts the sensor attached to the knee to measure the knee flexion angle. Here, the pressure signal intensifies in tandem with the increasing knee flexion angle, and when the knee is repeatedly bent to the same angle, the pressure signals remain largely unaltered, testifying to the sensor’s excellent stability. Figure 4g shows the pressure signals recorded by the sensor when the volunteer bends the elbow at 30°, 60°, and 90°. When the joint is in a relaxed state, the sensor is securely fastened to the joint under examination using medical adhesive tape. As the joint bends, the sensor experiences pressure and generates corresponding output signals. Crucially, the wrinkled structure of SWCNT/rGO-PDMS enables it to withstand the tensile forces exerted when the sensor is passively stretched during joint flexion, safeguarding against damage and endowing the sensor with the high sensitivity and stability requisite for human body monitoring applications.

### 3.4. Pressure Response and Distributed Testing of SWCNT/rGO-PDMS Under Minuscule Pressure

**Figure 5:**
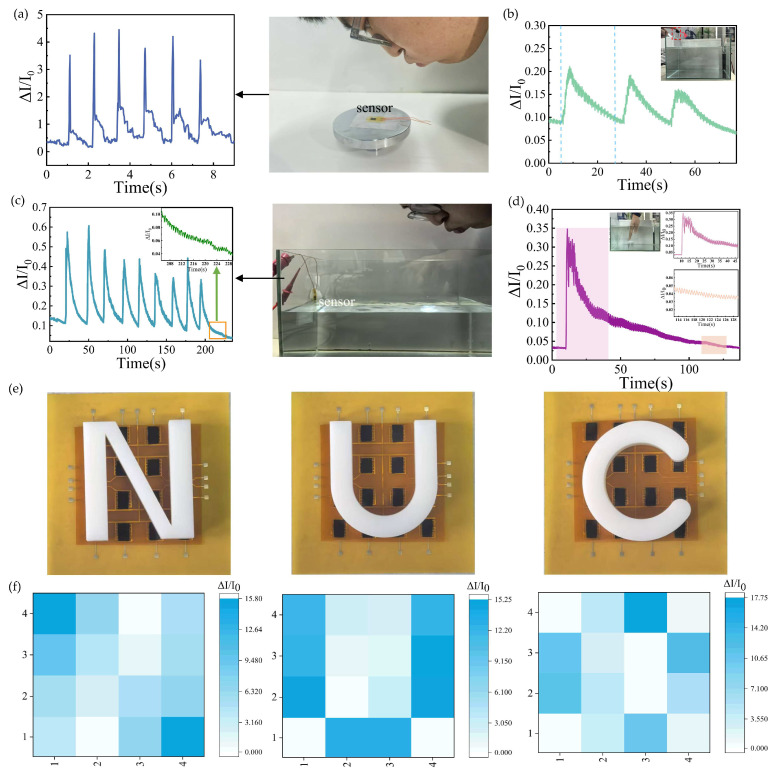
Minuscule pressure and distributed pressure testing. (**a**) The electrical response of the sensor when blown multiple times. (**b**) Image of the current corresponding to the glass bottle thrown into the water. (**c**) Current of the sensor when a volunteer blew air into the water. (**d**) Pressure response when a volunteer plunged a hand into the water and then swiftly withdrew. (**e**) Bright field of placing three different 3D-printed shapes (N, U, C) on the sensor array. (**f**) Heat map distributions of relative current variation of the sensor array with different letters on its surface.

In order to evaluate the response performance of the sensor under minute pressure, we conducted separate tests on the pressure response output of the sensor when exposed to air blowing and water wave impacts. Typically, the pressure generated by an average person blowing air hovers around 0.3–0.7 KPa. We secured the sensor onto the testing platform, and volunteers repeatedly blew on the sensor at a 45° angle above it. As illustrated in Figure 5a, the pressure response of the sensor with each puff of air exhibited a generally consistent magnitude trend. This implies that the sensor is proficient in monitoring the trifling pressure of exhaled air. Capitalizing on this feature, the sensor can be incorporated into masks to fabricate smart masks capable of tallying the number of human breaths. By gathering such data, it can offer valuable support for monitoring human health conditions. To further appraise the sensor’s efficacy in detecting minute pressures, we immersed the sensor in water and exerted pressure on it by inducing water surface undulations. The sensor was firmly affixed to the interior of a glass tank using waterproof tape. Figure 5b depicts the pressure responses of the sensor over multiple repetitions of dropping the same glass bottle into the water tank. Noticeably, the magnitudes of the pressure responses remained largely consistent throughout the three trials. Figure 5c showcases the output signal of the sensor when a volunteer blew air into the water, along with an enlarged view of the signal’s tail end. The pressure responses triggered by each bout of air blowing were essentially uniform, and the tail end of each pressure response manifested a regular waveform. The vibration of the water caused by blowing air can approximately demarcate the minimum excitation level to which the sensor can respond. Multiple air blowing tests have attested that the sensor retains high stability even under the pressure of infinitesimal water waves, boding well for its other potential applications in aquatic settings.

Figure 5d presents the pressure response of the sensor when a volunteer plunged a hand into the water and then swiftly withdrew it. It is evident that the moment the hand entered the water, the current spiked rapidly. As the hand was retracted, a chaotic waveform emerged. Once the hand was fully removed and the water waves in the tank were oscillating freely due to inertia, the response signal of the sensor also inclined to decline in an orderly fashion until it ultimately reverted to a stable state. These tests on minute pressure in water have spotlighted the sensor’s outstanding performance and remarkable stability in detecting minute pressure, corroborating the feasibility of its operation in aqueous environments. Given the sensor’s exceptional sensing traits in water, it can be worn on children or installed at the water level of park riverbanks. In the event of a child tumbling into the water, the ensuing water waves will impinge on the sensor, prompting it to generate a pressure response. Furthermore, by deploying sensors across different sections of the riverbank and integrating them with the Internet of Things, it becomes possible to institute a fall-into-water monitoring and alarm system. By leveraging the pressure responses from sensors in various areas, the location of the person who has fallen in can be promptly ascertained, hastening the arrival of rescue teams and enhancing the survival odds of those in peril. Figure 5e showcases the application of the SWCNT/rGO-PDMS sensor in conducting distributed tests of spatial pressure. We mounted the sensors onto array electrodes, thereby constructing a sensor array comprising 16 sensitive units. By augmenting the spacing between each sensing unit, we managed to mitigate the interference among them. Three distinct letters (N, U, C) were fabricated via 3D printing technology and subsequently positioned atop the sensor array. The array sensor exhibited an excellent response to spatial pressure. In the face of varying coverage areas, the sensor manifested different current magnitudes. When the currents of the array were visualized through pressure maps, it became patently clear that the three different letters could be discerned based on the weights depicted in the pressure maps shown in Figure 5f. This demonstrates that the sensor is capable of differentiating pressure distributions, suggesting that the SWCNT/rGO-PDMS sensor holds extensive application potential in the realm of Braille recognition.

### 3.5. High-Precision Recognition Based on AI

**Figure 6:**
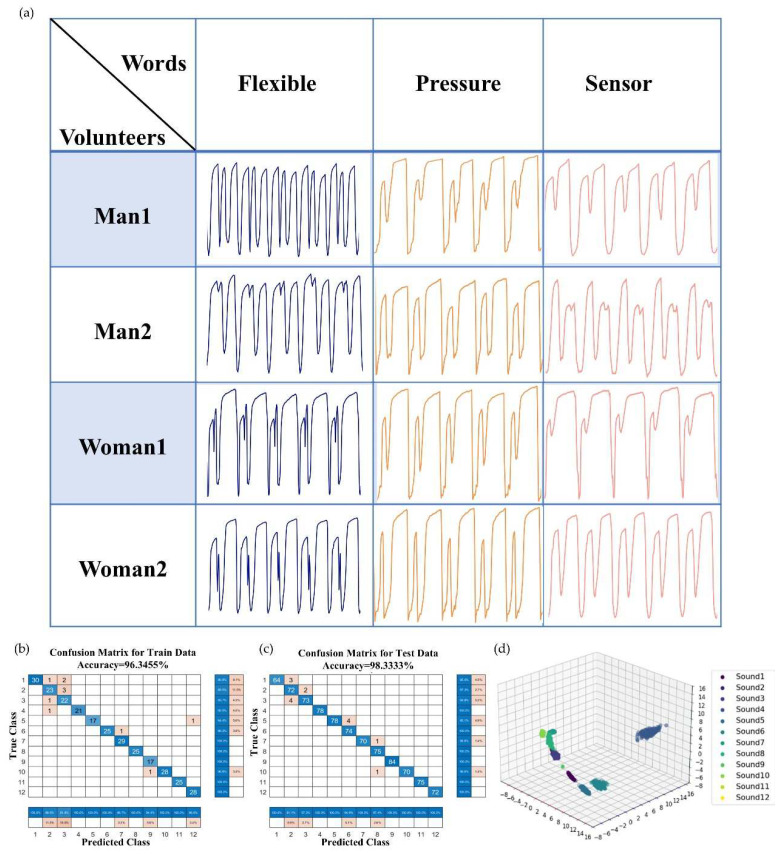
AI-based speech recognition. (**a**) Current curve of three words generated by four volunteers. (**b**) Confusion matrix for speech recognition of the training set. (**c**) Confusion matrix for speech recognition of the test set. (**d**) Clustering outcomes for the 12 data points set within the dataset.

The high stability and outstanding minute pressure detection capabilities of SWCNT/rGO-PDMS render it suitable for high-precision speech recognition applications. To further assess its practicality in this regard, the sensors were affixed just beneath the throats of four healthy adults, two of whom were male and two female. These volunteers were then instructed to read aloud three distinct words, namely “flexible”, “pressure”, and “sensor”. As the voice signals were being collected in Figure 6a, it was observed that when pronouncing “flexible”, the signals from volunteers of the same gender bore a resemblance to one another, while those from different genders exhibited marked disparities. When uttering “pressure”, the voice signals of all four volunteers were fairly similar, with only a slight variation in the pitch of the initial rising wave. In the case of “sensor”, the signals of three volunteers were highly alike, barring the second male volunteer. This divergence could be attributed to the varying stress patterns that different individuals placed on the word’s syllables. Machine learning algorithms were enlisted to classify the gathered data. In total, the sensor amassed 1200 sets of data, each set containing 93 characteristic points, which added up to 111,600 data points. The backpropagation neural network, a multi-layer feedforward type, is trained through error backpropagation. Of the collected data, 900 were designated as the training set for machine learning. The trainlm training regimen was employed to train the neural network, with the Sigmoid function serving as the activation function and the number of iterations set at 1000. The root mean square error of the training target was kept below 1 × 10^−9^. The confusion matrices for the training set and the test set are depicted in Figure 6b and Figure 6c, respectively. Ultimately, the recognition accuracies for the three words read by the four volunteers reached 96.3455% for the training set and 98.3333% for the test set. Figure 6d presents the clustering outcomes for the 12 data points set within the dataset. The visual inspection reveals that the majority of the sounds bear a resemblance to one another. Remarkably, the classification accuracy of the neural network algorithm attains values of 96.3455% and 98.3333%, respectively. Such results are ample evidence of the viability of integrating this sensor with the neural network approach. Thanks to the sensor’s precise micro-pressure measurement capabilities and the integration of AI technology, it finds utility in areas like dialect recognition. By discerning dialectal content and translating it into the corresponding standard language for feedback to users, it significantly mitigates the language barriers that individuals face when working or seeking medical treatment away from home.

Smart finger rings that rely on WiFi transmission boast the edge of being able to transmit data across long distances. This feature empowers them to play a pivotal role in medical examinations, human–computer interactions, and health management. In this study, SWCNT/rGO-PDMS, a signal acquisition circuit, and a wireless communication module were integrated to actualize the long-range wireless transfer of pressure signals.

**Figure 7:**
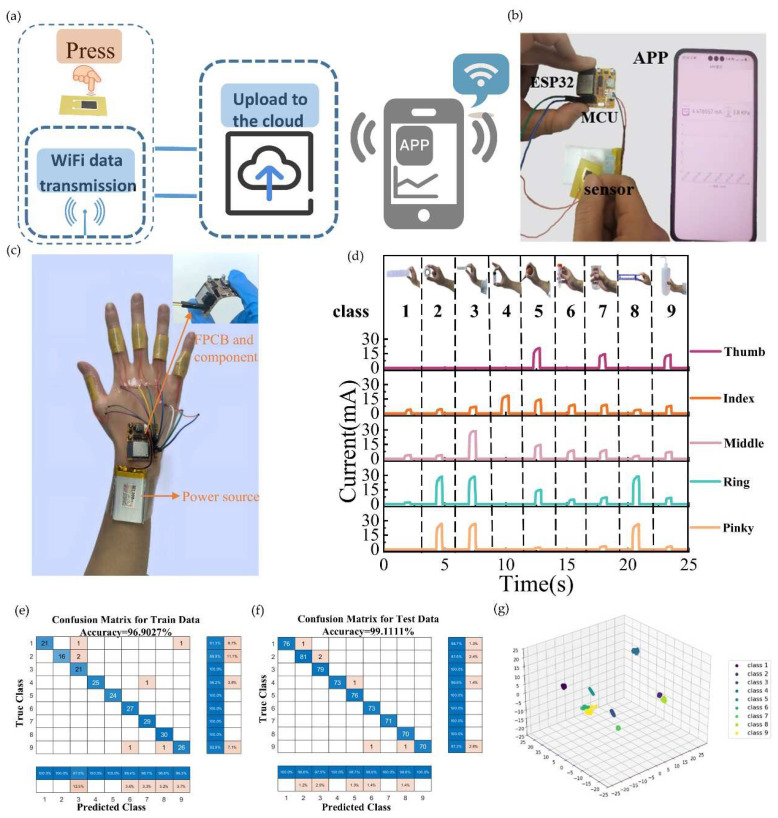
Wireless transmission-based object recognition and gesture recognition. (**a**,**b**) Acquisition system diagram and corresponding system. (**c**) Photograph of the grasping object recognition system, consisting of five smart finger rings, FPCB, and a battery. (**d**) Graph of the response of each finger on grasping different objects. (**e**) Confusion matrix diagram of the training set. (**f**) Confusion matrix diagram of the test set. (**g**) Cluster distribution of datasets processed by K-Means clustering algorithm.

Five sensors were incorporated into the joints of the five fingers. The signal acquisition module, by means of a flexible circuit board, was integrated and adhered to the back of the hand. Meanwhile, a 3.3 V thin battery was deployed to supply power to the entire system. By guaranteeing data collection and transmission while minimizing the number of connecting wires, the overall system was made more portable. The acquisition system gathered the current generated as each finger bent and dispatched the collected and processed data to the cloud via the WiFi module. Subsequently, the data in the cloud could be relayed to mobile phones, thereby enabling the remote monitoring of finger-bending states. Figure 7a and Figure 7b, respectively, illustrate the acquisition system diagram and corresponding system for sensor signal treatment. Based on the aforementioned wireless transmission system, the smart finger ring was devised. The rings in Figure 7c were fitted onto the finger joints to gauge the degree of bending of each digit and transmit the relevant data to mobile devices. The sensor signals captured when fingers grasped different objects are charted in Figure 7d. It is evident that fingers grasping disparate objects demonstrated varying degrees of flexion. The output current signals amassed during the grasping of different items were harnessed as the dataset for machine learning. Initially, the collected signal dataset underwent normalization. This step was crucial as it eradicated the dimensional influence among different features and enhanced the efficacy of the training model. For each set of data corresponding to different objects, 500 feature points were collected, and a backpropagation neural network was enlisted for object recognition. The dataset, which consisted of a total of 900 samples, was randomly partitioned, with three-fourths of it designated as the training set. The confusion matrices are presented in Figure 7e,f. The training set achieved an accuracy rate of 96.9027%, while the test set, when it came to nine objects, boasted an accuracy rate of 99.1111%. Figure 7g depicts the cluster distribution ensuing from the clustering of the dataset. Evidently, the majority of grasping gestures exhibit distinct characteristics, though a few clusters display some degree of overlap. Strikingly, the neural network algorithm achieves a classification accuracy rate exceeding 95%. This resoundingly attests to the superiority of integrating this sensor with the neural network algorithm in the realm of object grasping recognition. The smart finger ring has successfully accomplished the precise detection of diverse objects, epitomizing the high sensitivity of the sensor. This accomplishment holds great significance for remote human–computer interactions and robot pose recognition. Moreover, it can be applied to the remote diagnosis of traditional Chinese medicine pulses, effectively augmenting the efficiency of TCM diagnoses in remote regions.

## 4. Conclusions

In conclusion, the SWCNT/rGO-PDMS composite pressure sensor featuring a micro-wrinkled structure was fabricated via the heat shrinkage approach and PDMS coating. It has accomplished a broad pressure detection range spanning from 0 to 1150 KPa. Remarkably, this sensor demonstrates high stability in the face of temperature fluctuations, remaining impervious to temperature variations. Even after undergoing 18,000 cycles of testing, it sustains outstanding stability. Moreover, the sensor has verified its excellent capacity for minute pressure detection. It can adeptly pick up feeble physiological signals such as pulses and the gentle exhalation of air. Astonishingly, it can even detect the slightest vibrations of water waves, which renders it applicable for monitoring children who may have fallen into water. In addition, the sensor exhibits remarkable suitability for human body posture detection, be it joint flexion or throat vibrations. Owing to its high sensitivity, high-precision voice recognition has been achieved by means of AI algorithms, attaining a recognition accuracy rate of 98.3333%. This endows it with tremendous potential for identifying and differentiating dialects, thereby effectively dismantling communication barriers. Finally, a smart finger ring device has been devised. By leveraging machine learning algorithms and WiFi technology, it is capable of discerning nine distinct objects grasped by fingers, achieving a recognition rate of 99.1111%. This device holds great promise in human–computer interaction and is anticipated to find applications in areas like remote diagnosis in traditional Chinese medicine and fall-into-water monitoring.

## Data Availability

The data that support the findings of this study are available from the corresponding authors upon reasonable request.

## Supplementary Materials

The following supporting information can be downloaded at https://www.mdpi.com/article/10.3390/bios15020122/s1. Figure S1: Comparison of GO-SWCNT composite films before and after thermal shrinkage. Figure S2: Digital photographs of (a) graphene oxide, (b) SWCNTs, and (c) GO/SWCNT dispersion. Figure S3: Experimental setup for sensor response time testing. Figure S4: AutoCAD image (a) and physical image (b) of electrode array. Figure S5: Optical electron microscopy: (a) before heat shrinking; (b) after heat shrinking. Figure S6: Comparison of sensitivity under different GO/SWCNT contents; Figure S7: Design dimension of the interdigitated electrode; Figure S8: TEM images with the scale of 100nm and 50 nm of mixed dispersions of rGO/SWCNT; Table S1: The conductivity of pure SWCNT, rGO/SWCNT, GO/SWCNT, rGO and GO.
