# Supplementary material for: A Wide-Range, Highly Stable Intelligent Flexible Pressure Sensor Based on Micro-Wrinkled SWCNT/rGO-PDMS with Efficient Thermal Shrinkage
Source: Biosensors (Basel). 2025 Feb 19;15(2):122. doi: 10.3390/bios15020122 (PMC11852995; doi:10.3390/bios15020122)
Supplement: Supplementary file 1 [file biosensors-15-00122-s001.zip › biosensors-3455565-supplementary.pdf]

Supporting Information

Fabrication of Graphene Oxide

In this study, the Hummers method was employed to fabricate the Graphene Oxide[1]. Initially, the reaction flask was set up in an ice-water bath, into which 30 mL of concentrated sulfuric acid was carefully poured. A solid mixture composed of 2g of graphite powder and sodium nitrate (weight ratio of 3:1) was then added slowly to the concentrated sulfuric acid while stirring continuously to ensure uniform dispersion. Throughout this procedure, the temperature was strictly maintained below 20°C. During the extended stirring process, 5mg of potassium permanganate powder was added in several portions. After one to two hours, the temperature was elevated to 35°C, and stirring was continued for another three hours. Subsequently, 60mL of deionized water was added gradually and slowly, raising the temperature to 95°C. After stirring for 20minutes at this temperature, 350mL of 3wt% hydrogen peroxide was added to the solution, followed by continuous stirring. Finally, the reaction product was washed with dilute hydrochloric acid and deionized water successively, and then dried to obtain graphene oxide.

Supplementary Figures

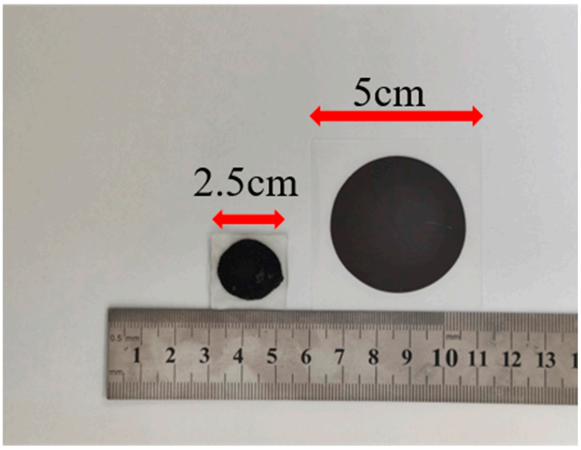

Figure S1. Comparison of GO-SWCNT composite films before and after thermal shrinkage.

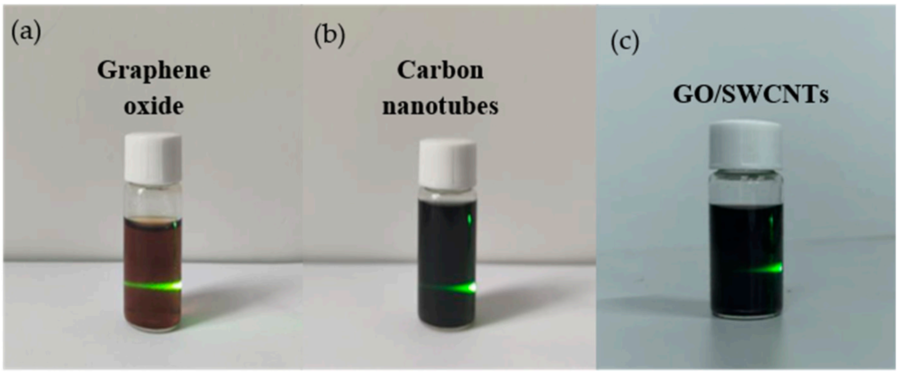

Figure S2. The digital photographs of (a) Graphene oxide, (b) SWCNTs, (c) GO/SWCNTs dispersions

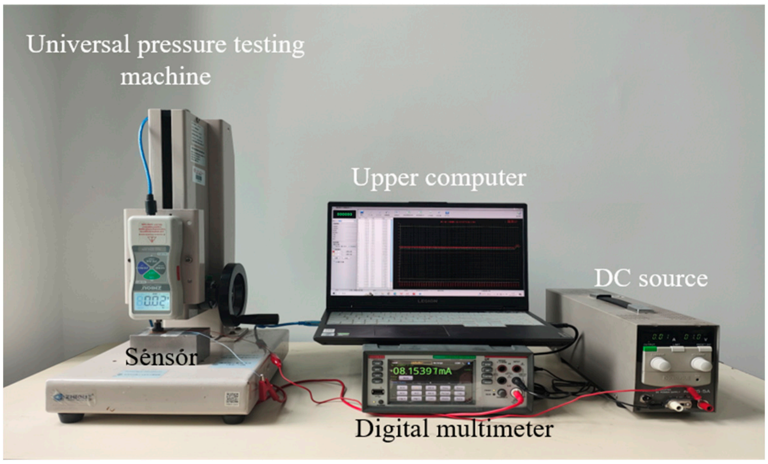

Figure S3. Experimental setup for sensor response time testing.

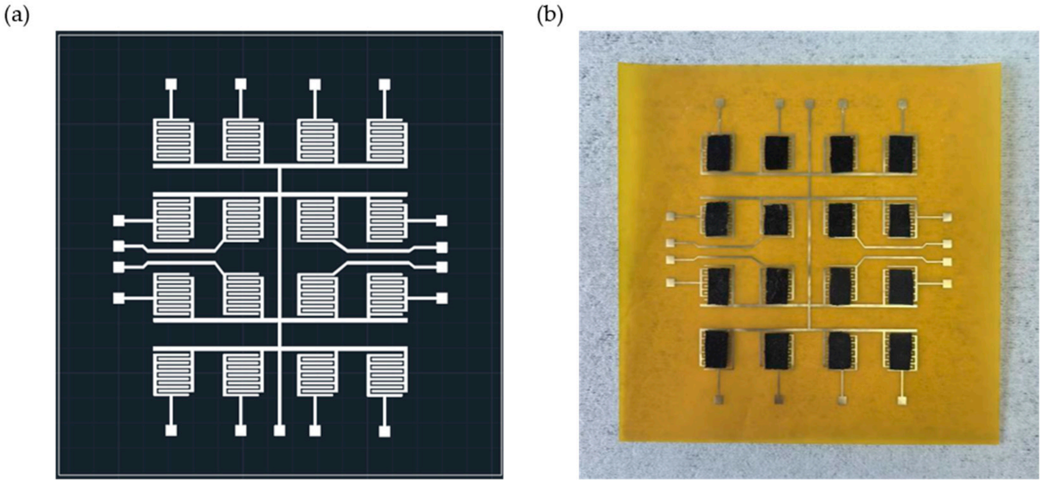

Figure S4. AutoCAD image (a) and physical image (b) of electrodes array.

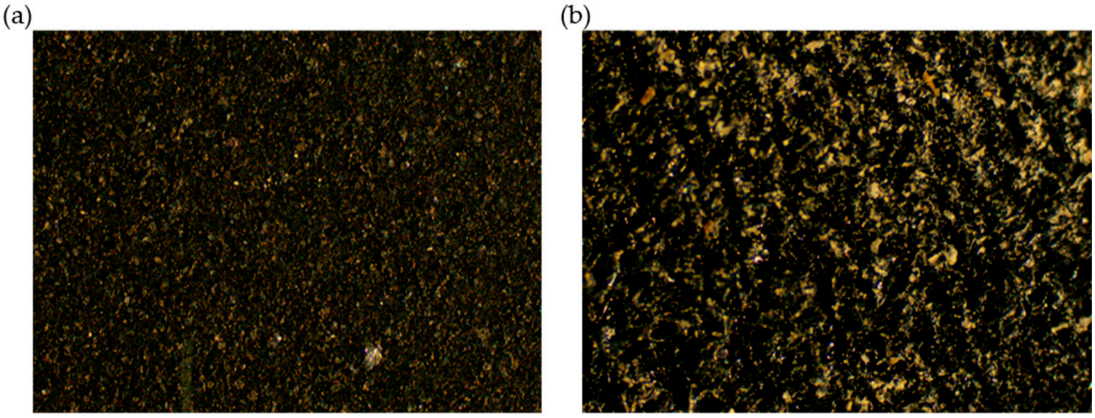

Figure S5. Bright-field images of micro-wrinkled structure (a)Before heat shrinking. (b)After heat shrinking.

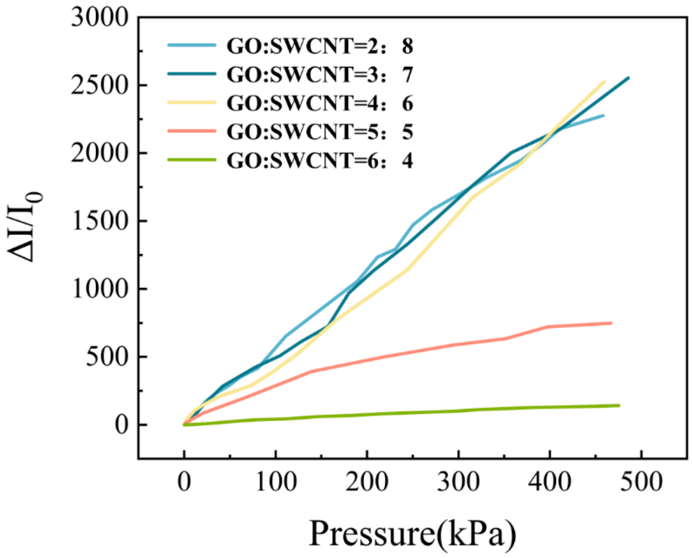

Figure S6. Comparison of sensitivity under different GO/SWCNT.

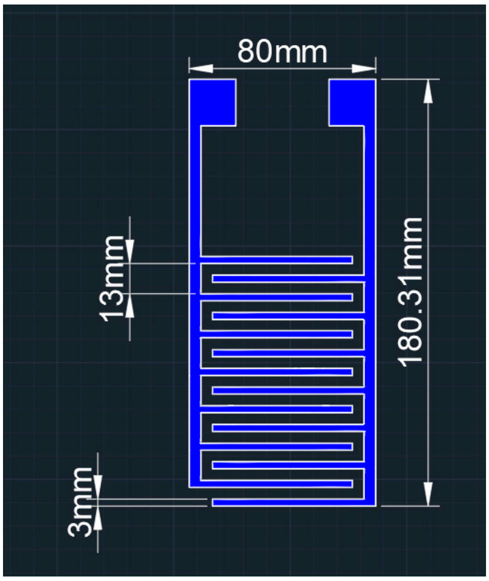

Figure S7. Design dimension of the interdigitated electrode.

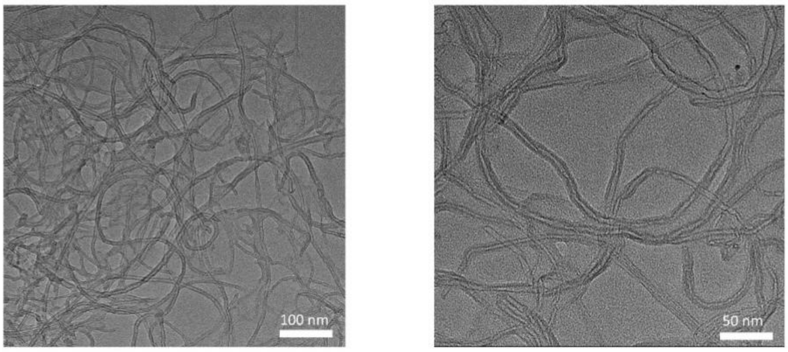

Figure S8. TEM images with the scale of 100nm and 50 nm of mixed dispersions of rGO/SWCNT.

**Materials**	SWCNT	rGO/SWCNT	GO/SWCNT	rGO	GO
**Conductivity**	91.74 s/cm	46.51 s/cm	37.88 s/cm	6.9×10^{-4} s/cm	2.76×10^{-4} s/cm

Table.S1. The conductivity of pure SWCNT, rGO/SWCNT, *GO/SWCNT, rGO and GO.*

Reference

1. Hummers Jr WS, Offeman REJJotacs. Preparation of graphitic oxide.1958;80: 1339-1339.
